# Integration and Disruption Effects of Shape and Texture in Haptic Search

**DOI:** 10.1371/journal.pone.0070255

**Published:** 2013-07-22

**Authors:** Vonne van Polanen, Wouter M. Bergmann Tiest, Astrid M. L. Kappers

**Affiliations:** Helmholtz Institute, Utrecht University, Utrecht, The Netherlands; Bielefeld University, Germany

## Abstract

In a search task, where one has to search for the presence of a target among distractors, the target is sometimes easily found, whereas in other searches it is much harder to find. The performance in a search task is influenced by the identity of the target, the identity of the distractors and the differences between the two. In this study, these factors were manipulated by varying the target and distractors in shape (cube or sphere) and roughness (rough or smooth) in a haptic search task. Participants had to grasp a bundle of items and determine as fast as possible whether a predefined target was present or not. It was found that roughness and edges were relatively salient features and the search for the presence of these features was faster than for their absence. If the task was easy, the addition of these features could also disrupt performance, even if they were irrelevant for the search task. Another important finding was that the search for a target that differed in two properties from the distractors was faster than a task with only a single property difference, although this was only found if the two target properties were non-salient. This means that shape and texture can be effectively integrated. Finally, it was found that edges are more beneficial to a search task than disrupting, whereas for roughness this was the other way round.

## Introduction

The efficiency of haptically searching for objects largely depends on the properties of the object and that of the irrelevant distractor objects surrounding the target object. Take the example of finding your phone in your bag. This would be an easy task, if the phone were the only item in the bag. With additional things in the bag, this task is more difficult, especially if the other objects resemble the phone. One can imagine that the more the phone differs from other objects (e.g. a pencil versus a calculator), the easier the task becomes. On the other hand, it also does matter whether the property of the object that is searched for can be easily perceived. Furthermore, the other items in the bag can also distract you from the task; for instance, feeling the bristles of a hairbrush is hard to ignore.

Search tasks can be used to investigate the neuronal processing of haptic perception. For instance, if a certain target can be found quickly, it is assumed that the processing of the target property is efficient and fast. On the other hand, if it takes longer to find a target, this target property is processed less efficiently. Also, there might be an interference of distractor items that are more easily noticed than the target. The example above illustrates some important points that seem to determine the performance in a search task: target identity, distractor (context) identity and the differences between target and distractors. In this study, we were interested in the contributions of the properties of the targets and distractors on haptic search performance. More specifically, the importance of target saliency, distractor disruptions and the integration of properties were of interest. Especially the integration of object properties in haptic perception is a subject of research that is rarely investigated. In the following sections the three main questions will be further explained.

### Target Saliency

To start with, it is evident that some targets are easier to find than others. If a target feature is immediately and almost automatically picked up from a scene, it is said this feature “pops out” [1]. Following this observation, the target feature is then believed to be relatively salient with respect to its context. Knowing more about features that are often salient can teach us more about what the basic properties, or primitives, in haptic perception are. These features may play an important role in the early recognition and exploration of objects [2]. In a search task, searching for a feature that is relatively salient with respect to its context is easy and can be done in a parallel way. This means that all items can be examined at once. This is in contrast to a serial strategy, where each item has to be explored separately to determine whether it is a target or not. Because of these differences in search strategies, in search tasks generally reaction times are measured, i.e. the time participants need to decide whether a target is present or not. When a serial strategy is used, the reaction times will increase with the number of items and therefore higher reaction times are observed than with the use of a parallel strategy when a large number of items need to be searched. In haptic search, the strategy can also be observed by looking at the explorations used by participants [3]; a single grasp may be sufficient to determine target presence, indicating parallel search. Alternatively, items may need to be felt one by one, which suggests a serial strategy. So, from the reaction times and the search behaviour, the saliency of a target property can be deduced. Previous research has revealed a number of salient haptic features: roughness [3], edges and vertices [4], temperature [5], movability [6] and hardness and softness [7] in active perception. It is important to note that for predicting the behaviour in a search task, the saliency of a target cannot be viewed without consideration of the distractors: a certain property in the target will not always pop out; this also depends on the context the target is in. For instance, when the target and distractors are alike, the target will be hard to find. However, this does not mean that the performance in a search task solely depends on the discriminability between the target and the distractors. In an asymmetric search a target property can be found easily among the distractors, but when their identity is reversed the task suddenly takes much more time. For example, a rough item is easily found among smooth ones, but not the other way round [3]. This illustrates that roughness is more salient than smoothness.

In that study [3], the pop-out of roughness was investigated by letting participants move their hand over a 2D-display with patches of sandpaper. In this 2D-setup, a lateral movement could be used, which is the optimal exploratory procedure (EP) for roughness perception [8]. However, in daily life we more often handle 3D objects and the exploratory behaviour might then be different. In addition, Van Polanen et al. [7] showed that the pop-out of a feature sometimes depends on the position of the distractors and the EP used. Therefore, it seems important to investigate whether roughness still pops out in the perception of 3D objects. It was not the intention of this study to investigate the roughness saliency in great detail, since this was already done in [3]. Note that we have used only a single number of items, which does not allow for the calculation of search slopes. This limits claims about pop-out and saliency. However, if roughness is more salient than smoothness, lower reaction times in the search for a rough target can be predicted with a large number of items, indicating that roughness is more salient than smoothness. Our first aim of this study was to investigate whether the search for a rough target is still quicker than the search for a smooth target if the items are 3D objects that can be freely manipulated in the hand.

### Salient Disruptions

A second important determinant of search performance is the identity of the distractors. In the original feature integration theory of Treisman [1], (salient) features were processed pre-attentively, in an automatic fashion. A bottom-up (stimulus-driven) form of perception was thus suggested. Although the theory has been challenged and revised since then (see [9], for a review) and a larger role for top-down processing has been suggested (e.g. [10]), in search tasks the search behaviour can be driven by the stimulus itself. Because features that are salient are easily and automatically perceived, they are also hard to ignore. In a search task, the perception signal of the target is the important signal. The signals of the distractors need to be ignored and treated as noise, since they are not searched for. However, this might be more difficult when in a task the distractors possess a feature that can be considered salient. When this task is compared to a search task where the distractors do not have this salient property, search efficiency might be disrupted, since the salient distractors are easily noticed. Possibly, such salient features in the distractors add a lot of noise, which might influence the performance by altering the signal-to-noise ratio of the target (signal). So, a salient feature in a distractor might then act as a disruption. This partly explains why the search for the absence of a feature (e.g. smooth among rough, stationary among movable) is much slower than the search for the presence of that feature. Moreover, even if (relatively) salient features are irrelevant for the task at hand, they might still disrupt performance. In a study by Panday, Bergmann Tiest and Kappers [11], participants showed different thresholds in perceiving the aspect ratio of rectangular blocks depending on the exploration strategy that was used. Higher thresholds and thus worse discrimination performance was found if participants freely explored the object instead of only touching the sides and avoiding the edges. The authors explained this by the disrupting effect of the salient edges of the blocks, which are only felt when participants slide their hand over the object.

In addition, it has been shown that salient features can bias perception. For instance, when participants haptically discriminate the volume of two objects, a bias is found when one object possesses an irrelevant but salient property: the salient object is judged larger or smaller than the other object without the salient property whereas they are actually equal in volume. This has been found for the object dimensions roughness, thermal conductivity and compliance [12].

This literature demonstrates the influence of salient features on discrimination thresholds. In this study, we were interested in how a salient feature could disrupt search performance when it is irrelevant for the task. In search tasks, the difference between target and distractors is much larger than the just noticeable difference, but it might still affect the performance in the task.

### Integration

Intuitively, it may seem that the more the target differs from the distractors, the easier the search task becomes. This has been confirmed by studies that varied the range in which target and distractor differed on a single property [3], [7]. However, it is unclear how the number of available properties influences the performance in a search task. In fact, there is very little research into the integration of object properties in haptic perception. The studies that investigated haptic integration mainly focussed on the weighting of different cues for the percept of a single property [13]–[18]. It is possible that if a target differs in two properties from the distractor, the performance is better than both searches where only one of the properties is different. In other words: the two cues are integrated to improve the performance. A cue is here defined as a feature that is different between the target and the distractors and can thus be used to discriminate between the two. A study by Klatzky, Lederman and Reed [19] demonstrated that object properties could be integrated in a classification task. Participants were much faster in sorting objects when two properties defined a category than if only one property specified the group to which the object belonged. Furthermore, if objects could be sorted according to two properties and later one was removed, participants’ reaction times also increased.

On the other hand, perception might also be based on the best cue available. In this way, the search is simplified to a single-feature search task. This might be advantageous if there is a pop-out of one of the features, but the other feature is much less salient. The perceptual system might then focus on the most salient cue available.

However, the addition of a discriminating feature might not always be advantageous because this feature might also disrupt performance. To be more specific, if one has to search for a rough item among smooth distractors this is an easy task. When an extra cue is added to this task by making the target a rough sphere among smooth cubes, the target and distractors do not only differ in roughness but also in shape. This extra cue might improve search performance because of cue integration. On the other hand, the salient edges that are now present in the distractors might also disrupt performance. The question then is whether the advantage of the cue outweighs the disadvantages of the disruption or that they balance out each other. The final aim of this study was therefore to investigate how search performance is influenced by the presentation of more than one feature difference between target and distractor, whether this information can be integrated and how this depends on possible disruptions.

To investigate all the questions raised above – the influence of target saliency, disruption and integration − we set up several search conditions using four different stimulus types. These stimuli were the combinations of a roughness and a shape (edge) property: a rough sphere, a smooth sphere, a rough cube and a smooth cube. We chose roughness and edges as features to investigate for the following reasons. First, these two properties can be very salient and secondly, they show search asymmetries. Plaisier et al. [3] found that participants were faster in the search for a rough target among smooth distractors than the other way round. In a similar study, they found that the search for cubes among spheres was faster than vice versa [4]. Each possible combination of target and distractor pair was made and selective comparisons between the conditions will give insights into the contributions of the cues and disruptions to the search behaviour.

With respect to target identity, it was expected that a rough item among smooth items would be quicker to find than vice versa. Similarly, a cube was expected to be quicker to find among spheres than the other way round. If an irrelevant disruption were added to these search tasks, by using cubes instead of spheres in the roughness search tasks and rough items instead of smooth ones in the shape search tasks, the search was expected to be slower. Regarding the questions about integration, it was hypothesized that when two cues are available to distinguish the target from the distractors, the search would be faster than when the target would only differ on a single property. Finally, the balance of cues and disruptions was evaluated by comparing a condition to another where both a cue and a disruption were added. If performance improved, the cue would weigh heavier, whereas the disruption would weigh heavier in the case of a decrease in performance. With no change in performance, the cue and disruption would outbalance each other.

## Methods

### Participants

Ten participants (5 females) were recruited for the experiment, with a mean age of 24±3 years. They were all right-handed according to Coren’s test [20] and used their dominant hand in the experiment. They gave their written informed consent prior to the experiment and were paid for their contribution. The study was approved by the Ethics Committee Human Movement Sciences (ECB).

### Apparatus

**Figure 1:**
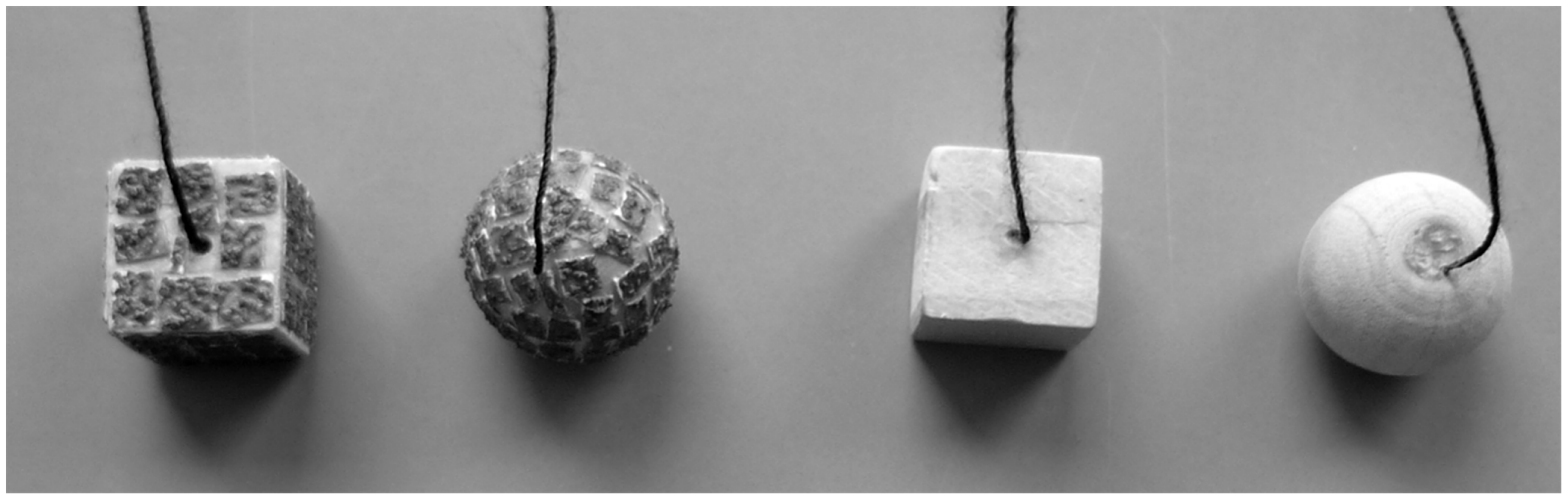
Examples of the stimuli. From left to right: rough cube, rough sphere, smooth cube, smooth sphere.

Four kinds of stimulus items were used in the experiment: rough spheres, smooth spheres, rough cubes and smooth cubes (Figure 1). The stimuli were all made of wood and weighed about a gram. The spheres were beads (Pipoos) with a diameter of ∼15 mm. The cubes had an edge length of ∼12 mm, making the spheres and cubes approximately equal in volume (spheres: 1.8 cm^3^, cubes 1.7 cm^3^). The rough stimuli were created by gluing small pieces of sandpaper (Bosch, P60) on the stimuli. The edges of the sandpaper were of a very small scale and were perceived as “something rough”; therefore, they were not confused with the large-scale edges of the cubes. A piece of string was glued to each stimulus and they were grouped in bundles of 7 items. Since greater differences between conditions can be expected in larger set sizes, a number of 7 items was chosen, which was the largest number that could fit comfortably in the hand. Each of the four stimulus types could be used as a target. Four bundles did not contain a target (target-absent bundles). Others had one target and 6 distractors, making one target-present bundle for each condition (see Table 1).

**Table 1:** A basic overview of the conditions.

condition	name	target		distractors		roughness	shape	# cues	# disruptions
1	Rough1	•	Rough sphere	○	Smooth sphere	×		1	0
2	Rough2	▪	Rough cube	□	Smooth cube	×		1	1
3	Smooth1	○	Smooth sphere	•	Rough sphere	×		1	1
4	Smooth2	□	Smooth cube	▪	Rough cube	×		1	2
5	Cube1	□	Smooth cube	○	Smooth sphere		×	1	0
6	Cube2	▪	Rough cube	•	Rough sphere		×	1	1
7	Sphere1	○	Smooth sphere	□	Smooth cube		×	1	1
8	Sphere2	•	Rough sphere	▪	Rough cube		×	1	2
9	Rough&cube	▪	Rough cube	○	Smooth sphere	×	×	2	0
10	Smooth&sphere	○	Smooth sphere	▪	Rough cube	×	×	2	2
11	Rough&sphere	•	Rough sphere	□	Smooth cube	×	×	2	1
12	Smooth&cube	□	Smooth cube	•	Rough sphere	×	×	2	1

Note: the symbols indicate the stimuli, where squares stand for cubes and circles for spheres; filled and open symbols represent rough and smooth stimuli, respectively. The columns ‘roughness’ and ‘shape’ specify whether the target and distractor differed with respect to that feature. The last two columns indicate the number of available cues and disruptions, respectively.

**Figure 2:**
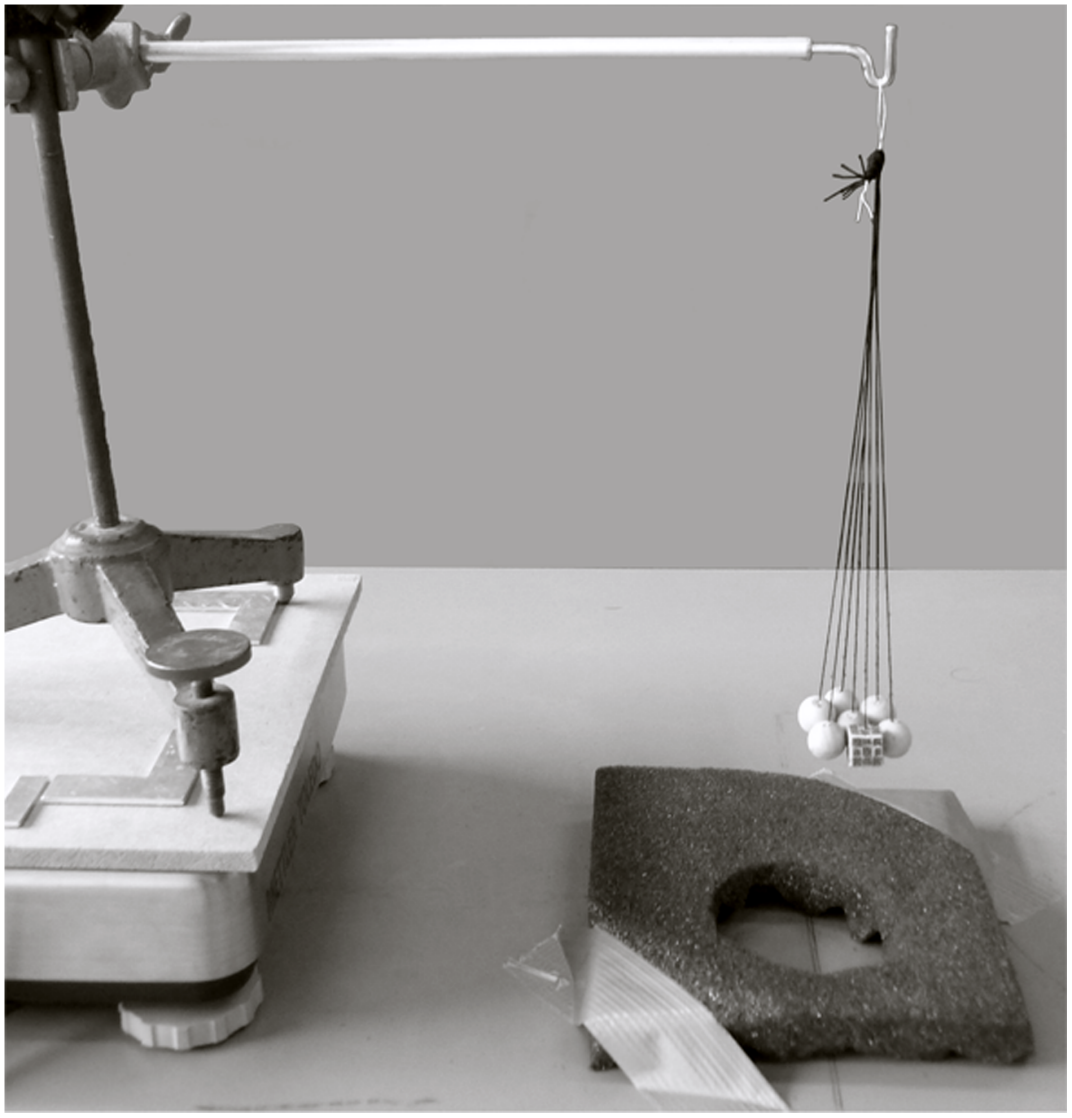
Experimental set-up. A bundle of items (one rough cubical target amongst smooth spherical distractors) hang above a resting cushion.

The experimental set-up was similar to that in Van Polanen et al. [7]. A tripod was placed on a weighing scale (Mettler Toledo SPI A6) as shown in Figure 2. A bundle of stimuli could be hung onto a hook attached to the tripod. The reaction time was measured from the moment the participants touched the items; a weight change induced by the participant lifting the bundle started the clock. The end of the reaction time was measured by a vocal response, recorded with a head-set placed on the participants head. The sample frequency of the reaction time was 100 Hz. The weighing scale had a delay of 90±20 ms (as measured by [7]), which was added to the raw data.

### Task and Procedure

The stimuli were combined as target-distractor pair in all possible combinations, resulting in the 12 conditions as shown in Table 1. The names in conditions 1–8 were chosen based on the target property that was to be searched for. The conditions ending with “1” contain no possible irrelevant disruption, whereas conditions ending with “2” do. Conditions 9–12 all have two property differences between the target and distractor and are named according to the shape and texture of the target. In the analysis (see below), specific conditions were compared to answer the specific research questions as described in the Introduction.

In each condition, the task was basically the same. Blindfolded participants had to determine whether a target was present or not and indicate this by calling out ‘yes’ or ‘no’. They were told there could be a bundle with seven items that were the same (target-absent trial), or a bundle with six items that were the same and one different target (target-present trial). Before the start of each condition, the target and distractor type were shown and it was explicitly stated whether the target and distractors differed in roughness, shape or both. Before the start of the trial, participants put their flat hand, with the palm up, upon the resting cushion underneath the bundle (Figure 2). They were instructed to lift their hand and initially grasp the bundle, but could then freely manipulate the items or drop them out of their hand. It was told that they should only do that if they thought this was the best strategy. It was stressed that it was important to answer as fast as possible, but also to make as few mistakes as possible. They received feedback whether their answer was correct. For each condition, at least 20 practice trials were performed. More trials were taken until 10 trials were answered correctly in a row, up to a maximum of 35 practice trials. During the practice trials, participants were encouraged to try out different strategies in order to find one that was fast, but did not lead to mistakes.

For each condition, 40 trials were performed, of which half contained a target. The target-present and target-absent trials were presented in a random order. The location of the target was not systematically controlled, but it was located at different positions in the bundle. However, by manipulating the items in the hand, participants could change the position of the target as well. Incorrect answers were repeated at the end of the condition. The order of the conditions was roughly counterbalanced between participants by using a balanced Latin square. In this way, no sequence of two conditions occurred more than once. Participants carried out three conditions in one session of about 1 hour. The four sessions were separated by at least a few hours and no more than two sessions were performed on a single day.

### Analysis

Four trials (<0.1%) were removed from analysis due to measurement errors. Only correctly answered trials were included in the analysis (but note that the number of errors was recorded). Mean reaction times were calculated for each condition and for target-present and target-absent trials separately. Outliers (0.6%) that fell above or below 3 standard deviations from the mean were removed from further analysis.

Furthermore, it was scored whether participants dropped items out of their hand. The proportion of trials where this happened was calculated. This measure of exploration strategy has been used previously in studies with similar tasks, e.g. [4], [7]. Also the proportion of errors was calculated. In this case, a percentage was calculated as the number of errors divided by the total number of correct trials+the number of errors.

A 12 (condition)×2 (target presence) repeated measures Analysis of Variance (ANOVA) was conducted on the mean reaction times. The α-value was set at 0.05. Post-hoc tests were performed with paired-samples *t*-tests with a Bonferroni correction. Planned comparisons were made, that is, conditions were only compared when relevant for the research questions as described in the Introduction. The comparisons between conditions to be made are listed in Table 2. The 30 comparisons in this table were made for target-present and target-absent trials, making 60 comparisons in total. In addition, for each of the twelve conditions target-present and target-absent trials were compared. This makes a total of 72 comparisons and the α-value was divided by this number. Conditions were considered different if they differed when pooled over target-present and target-absent trials. Note that this means that conditions do not have to differ on both target-present and target-absent trials separately.

**Table 2:** Results from the planned comparisons for the reaction times.

	First condition				Second condition			
**saliency**	•	○	rough1	↔	○	•	smooth1	**
	□	○	cube1	↔	○	□	sphere1	*
**disruption**	•	○	rough1	↔	▪	□	rough2	**
	○	•	smooth1	↔	□	▪	smooth2	
	□	○	cube1	↔	▪	•	cube2	*
	○	□	sphere1	↔	•	▪	sphere2	
**integration**	▪	○	rough&cube	↔	•	○	rough1	
	▪	○	rough&cube	↔	□	○	cube1	
	•	○	rough1	↔	□	○	cube1	
	•	□	rough&sphere	↔	▪	□	rough2	
	•	□	rough&sphere	↔	○	□	sphere1	
	▪	□	rough2	↔	○	□	sphere1	
	○	▪	smooth&sphere	↔	□	▪	smooth2	**
	○	▪	smooth&sphere	↔	•	▪	sphere2	**
	□	▪	smooth2	↔	•	▪	sphere2	
	□	•	smooth&cube	↔	○	•	smooth1	*
	□	•	smooth&cube	↔	▪	•	cube2	
	○	•	smooth1	↔	▪	•	cube2	
**balance**	▪	□	rough2	↔	▪	○	rough&cube	**
	□	▪	smooth2	↔	□	•	smooth&cube	**
	▪	•	cube2	↔	▪	○	rough&cube	**
	•	▪	sphere2	↔	•	□	rough&sphere	
	○	•	smooth1	↔	○	▪	smooth&sphere	*
	•	○	rough1	↔	•	□	rough&sphere	
	□	○	cube1	↔	□	•	smooth&cube	**
	○	□	sphere1	↔	○	▪	smooth&sphere	
	▪	□	rough2	↔	▪	•	cube2	
	□	▪	smooth2	↔	□	○	cube1	**
	•	○	rough1	↔	•	▪	sphere2	**
	○	•	smooth1	↔	○	□	sphere1	

Note: Comparisons are sorted according to the research question of interest. Before each condition name the target and distractor type are illustrated (circles: spheres, squares: cubes; filled: rough item, open: smooth item). Asterisks indicate significant differences between the conditions pooled over target presence and target absence (*p<0.05, **p<0.01).

Finally, to investigate the relative contribution of the two cues, roughness and shape, and the disruptive nature of both, a linear regression was performed on the data. In this way, the cues and disruptions can be included in a model to describe the search performance. In Table 1, the cues that are available to distinguish between the target and distractors are listed for each condition. In the model, the presence of a roughness cue (*c_r_*) or shape cue (*c_s_*) was scored as 1, or 0 for its absence. Furthermore, the presence of a salient distractor property was included for roughness (*d_r_,* rough distractor) and for shape (*d_s_,* cube distractor with salient edges and vertices) as 1 or 0. A weighted sum of these four parameters (*c_r_*, *c_s_*, *d_r_* and *d_s_*) and a constant (*C*) was fitted to the reaction times using the following equation:


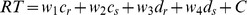
(1).

In this equation the ones and zeroes were inserted for the parameters and a linear fit was made to obtain a value for the four weights (*w_1…4_*) and the constant. *RT* is here the reaction time that is calculated by the model. The reaction times that were fitted were averaged over participants and over target presence. The latter was done because no apparent differences were found between target-present and target-absent fits, i.e. the weights of the parameters were relatively similar.

## Results

First, the main results of the ANOVA on the reaction times will be briefly described. Next, separate conditions are compared individually to answer the different research questions about target salience, salient disruptions and integration. Results for these comparisons are presented separately and are also summarized in Table 2. Last, the results for the number of errors and the search behaviour will be described.

### Reaction Times

**Figure 3:**
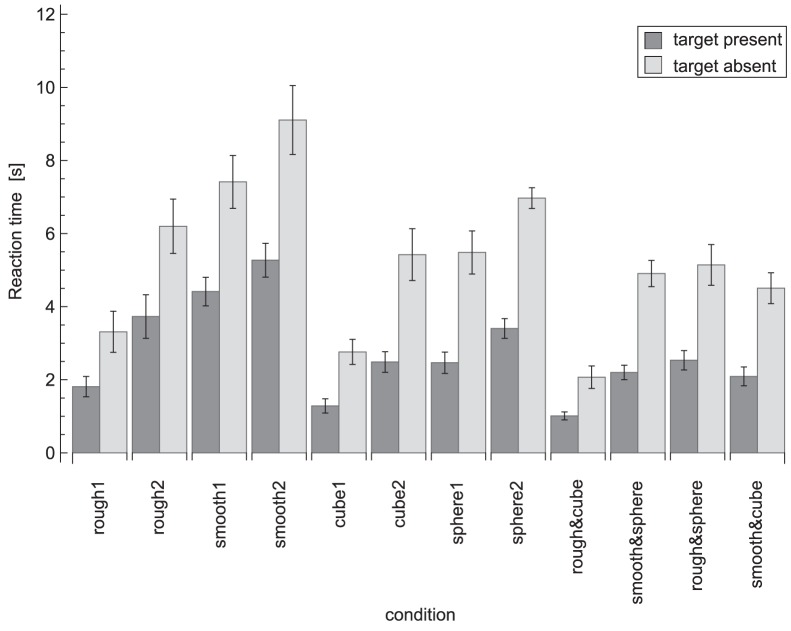
Reaction time data for all conditions. Dark grey bars indicate target-present trials and light grey bars target-absent trials. Error bars represent standard errors.

**Figure 4:**
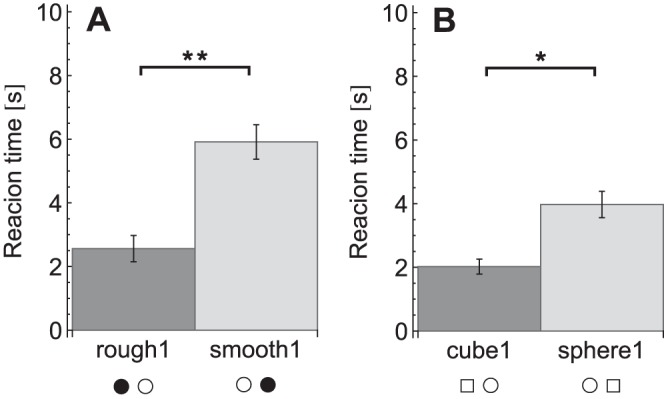
Reaction times for the target saliency comparisons. A: Roughness comparison. B: Shape comparison. Reaction times are pooled over target-present and target-absent trials. Legends indicate the conditions, where each symbol pair stands for the target and the distractors in that condition as explained in Table 1. *p<0.05, **p<0.01.

**Figure 5:**
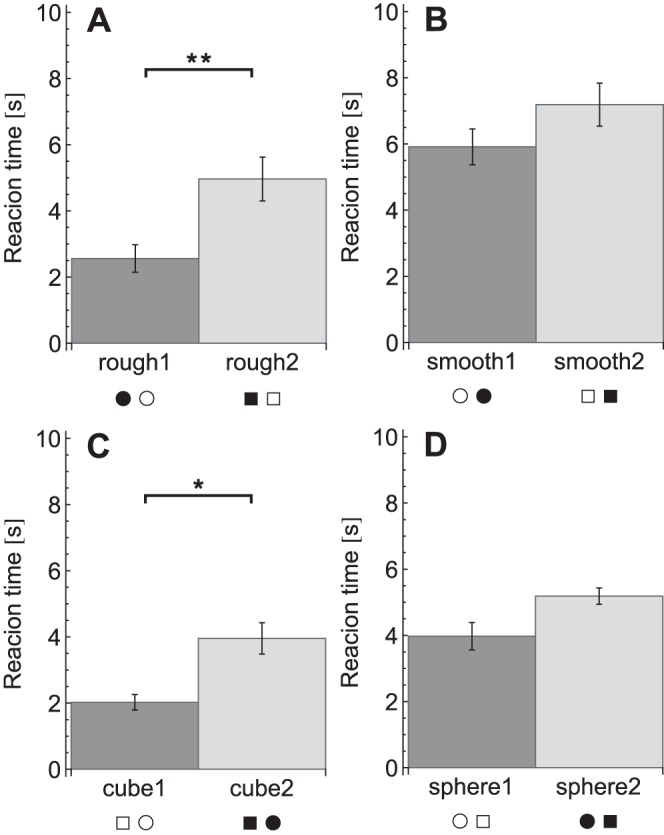
Reaction times for the salient disruption comparisons. A: Edge disruption on rough target. B: Edge disruption on smooth target. C: Roughness disruption on cube target. D: Roughness disruption on sphere target. Reaction times are pooled over target-present and target-absent trials. Legends indicate the conditions, where each symbol pair stands for the target and the distractors in that condition as explained in Table 1. *p<0.05, **p<0.01.

**Figure 6:**
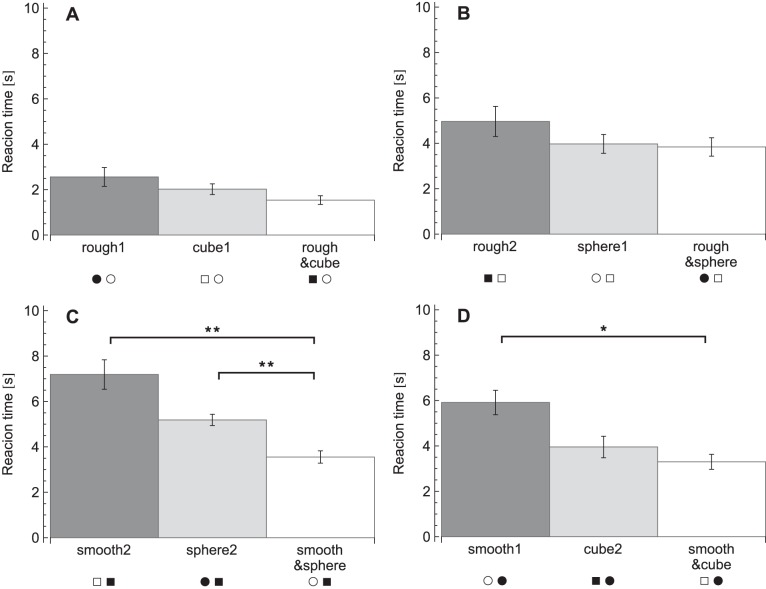
Reaction times for the integration comparisons. A: Comparisons of smooth sphere distractors. B: Comparisons of smooth cube distractors. C: Comparisons of rough cube distractors. D: Comparisons of rough sphere distractors. Reaction times are pooled over target-present and target-absent trials. Legends indicate the conditions, where each symbol pair stands for the target and the distractors in that condition as explained in Table 1. *p<0.05, **p<0.01.

**Figure 7:**
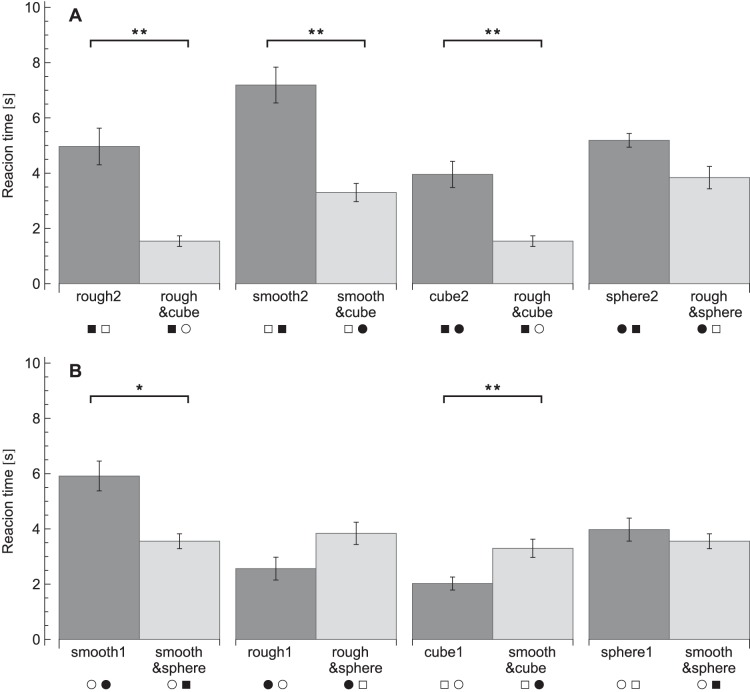
Reaction times for the balance comparisons. A: Comparisons with a cue added and disruption removed. The two comparisons on the left have a shape cue added, the two on the right have a roughness cue added. B: Comparisons with a cue and a disruption added. The two comparisons of the left have a shape cue added, the two on the right have a roughness cue added. Reaction times are pooled over target-present and target-absent trials. Legends indicate the conditions, where each symbol pair stands for the target and the distractors in that condition as explained in Table 1. *p<0.05, **p<0.01.

**Figure 8:**
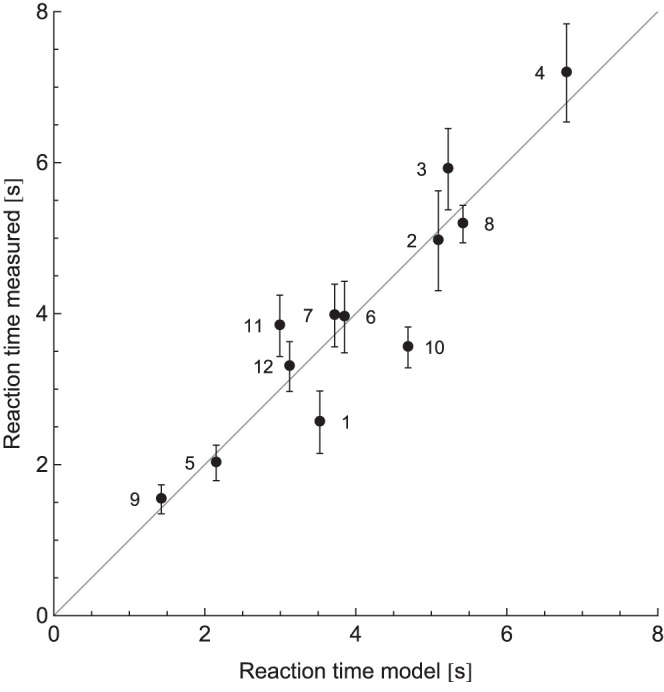
The measured reaction times against the calculated reaction times of the model. Error bars represent standard errors. The numbers next to the data points indicate the condition.

The reaction times for each condition are plotted in Figure 3. A repeated measures ANOVA on the reaction times revealed effects of condition (*F_11,99_* = 28, *p*<0.001), target presence (*F_1,9_* = 147, *p*<0.001) and an interaction between condition×target presence (*F_11,99_* = 6.5, *p*<0.001). The effect of target presence indicated that participants were faster in target-present trials (target present: 2.7±0.8 s, target absent: 5.3±1.3 s). When the conditions were analysed separately to investigate the condition×target presence interaction, reaction times were found to be significantly lower in target-present trials than target-absent trials, except in the three fastest conditions (rough1, cube1 and rough&cube), which showed no significant difference. The significant differences between the conditions for the planned comparisons are listed in Table 2 and figures 4, 5, 6, 7, 8.

Note that for reasons of simplicity, in figures 4, 5, 6, 7, 8 below the reaction times are shown averaged between target-present and target-absent conditions, whereas the statistical analysis is performed with those reaction times separate.

### Target Saliency

First, it was investigated whether roughness and edges could be relatively salient features. The compared conditions and results are shown in Figure 4. Results indicated that the conditions in which only a single cue was present showed a search asymmetry both for roughness and for shape. For these asymmetries, the conditions in which no irrelevant disruption was present were compared. For roughness, the search for a rough sphere among smooth spheres (rough1) was compared to the search for a smooth sphere among rough spheres (smooth1). Post-hoc tests revealed a significant difference, with smaller reaction times for rough1 (target present: *p*<0.001, target absent *p* = 0.002). With respect to shape saliency, the conditions with the search for a sphere among cubes (sphere1) and the search for a cube among spheres (cube1) were compared. Results indicated that reaction times were significantly shorter in cube1 in target-present trials (*p* = 0.005).

So, rough spheres were found faster than smooth spheres and the search for smooth cubes was faster than the search for smooth spheres.

### Salient Disruptions

The second question was whether salient edges and roughness could also disrupt performance when they were irrelevant for the task. To see if the edges disrupted performance in a roughness search task, the conditions with and without edges were compared: rough1 was compared to rough2 and smooth1 to smooth2 (see Figure 5A–B). In the search for a rough item among smooth, there was a difference between rough1 and rough2 (target present: *p* = 0.038, target absent: *p* = 0.001), where reaction times were higher in rough2 (with edges). None of the post-hoc tests showed significant differences between the two searches for a smooth item among rough items, smooth1 and smooth2 (with edges).

Likewise, to investigate the influence of roughness on the shape search tasks, cube1 was compared to cube2, as well as sphere1 to sphere2 (Figure 5C–D). In searches for a cube among spheres, cube1 (without roughness) gave significantly lower reaction times than cube2 (with roughness) in target-present trials (*p*<0.001). Similar to the smooth target conditions, no significant differences were found between sphere1 and sphere2 in any of the post-hoc tests.

To sum up, with the presence of a disruptive property, reaction times increased in the search for a rough or cubical target, but not for a smooth or spherical target.

### Integration

For the questions about integration and balance of multiple target properties, two sets of comparisons were made. First, to investigate the influence of the addition of a cue and integration of the two cues, conditions with the same kind of distractors were compared, as illustrated in Figure 6. Secondly, to examine the balance between disruption and cue integration, conditions with the same kind of target were compared (Figure 7). It was reasoned that if target and distractors would both differ in comparisons, too many (unknown) variables might be of influence and that would make the interpretation too difficult and uncertain.

The four integration conditions (8–12) were compared to the two single-cue conditions that shared the same distractors. It was hypothesized that if an integration condition showed increased performance compared to both single-cue conditions, then the two cues should be integrated. To start with the conditions with smooth spheres as distractors, rough&cube did not differ significantly from rough1 or from cube1 in any of the post-hoc tests (Figure 6A). Also, no effects were seen when comparing conditions with smooth cubes as distractors: the rough&sphere condition showed no significant reaction time differences when compared to rough2 or sphere1 (Figure 6B). In contrast, when comparing conditions with rough cubes as distractors, a significant difference between smooth&sphere and smooth2 for target-present trials (*p* = 0.002) and between smooth&sphere and sphere2 for target-absent trials (*p* = 0.006) was found (Figure 6C). Lastly, when comparing conditions with rough spheres as distractors, the smooth&cube condition showed no significant difference from cube2, but was different from smooth1 (Figure 6D). Reaction times were higher in smooth1 than smooth&cube only if a target was present (*p* = 0.017). No significant differences were found between the conditions that had the same distractors, but both only contained a single cue. Altogether, only the combination of a smooth sphere as a target gave lower reaction times than both searches with only a smooth target and only a sphere as target.

In the second set of comparisons to examine the balance of cues and disruptions, the target was kept constant. Comparisons could again be divided into two situations, depending on whether a disruption was added or removed. First, in the situations in which a cue was added and a disruption was removed, reaction times usually decreased (see Figure 7A). Reaction times were lower for rough&cube compared to rough2 (target present: *p* = 0.032, target absent: *p* = 0.008) and also when compared to cube2 (target present: *p* = 0.019, target absent: *p* = 0.040). Reaction times were also lower in smooth&cube compared to smooth2 (target present: *p* = 0.001, target absent: *p* = 0.041). In contrast, in the comparisons of rough&sphere with sphere2 no significant effects were observed. In sum, with the addition of a cue and the removal of a disruption, usually lower reaction times were found.

Secondly, there were a few situations in which a cue was added in combination with a disruption (Figure 7B). For the smooth&sphere condition, a significant difference with smooth1 was found in target-present trials, where smooth&sphere gave lower reaction times (*p* = 0.013). On the other hand, reaction times were higher in smooth&cube than cube1 (target present: *p* = 0.006, target absent: *p* = 0.029). So, the addition of shape decreased reaction times, whereas the addition of roughness increased the reaction time. However, there were also two cases in which no differences were found. When smooth&sphere was compared to sphere1, no significant effects were observed. Also no significant differences were found when rough&sphere was compared to rough1.

When the single-cue conditions were compared, also some differences were found (not shown in Figure 7). Smooth1 showed higher reaction times than sphere1 for target-present trials only (*p* = 0.020). Rough1 was different from sphere2 when the target was absent (*p* = 0.005), where reaction times were lower in rough1. Smooth2 did differ significantly from cube1, with higher reaction times for smooth2 (target present: *p*<0.001, target absent *p* = 0.012).

### Model

To investigate the contributions of the two cues and disruptions, a linear regression was fit to the mean reaction time data. The model fitted the data well, with an *R* ^2^ of 0.87. The measured reaction times are plotted against the calculated reaction times from the model in Figure 8. The fitted weight of each parameter in the model is displayed in Table 3. Immediately apparent is that the roughness cue was not significant. For the other parameters and the constant, *p*<0.01. The cues (*c_r_* and *c_s_*) have a negative value and thus shorten the reaction time. The disruptions (*d_r_* and *d_s_*) have a positive value and thereby increase the reaction time.

**Table 3:** The weights for each parameter in the linear regression model of Equation (1).

parameter	weights (s)
*c_r_*	−0.73
*c_s_*	−2.1**
*d_r_*	1.7**
*d_s_*	1.6**
constant	4.3**

Note: *c_r_* and *c_s_* are the cues for roughness and shape, respectively; *d_r_* and *d_s_* are the disruptions for roughness and shape, respectively.

** *p*<0.01.

### Errors

The percentage of errors in each condition is shown in Table 4. As can be seen, almost no errors are made in the target-absent trials, whereas a number of mistakes are made in target-present trials. This means it is more likely to miss a target, than to perceive one that is not there, as is typical in search tasks. Furthermore, it is clear that some conditions are more difficult than others, as more errors are made in these conditions (e.g. smooth2).

**Table 4:** Percentage of errors in each condition, with target-present and target-absent trials listed separately.

condition		Target Present (%)	Target Absent (%)
1	rough1	3	0
2	rough2	6	0
3	smooth1	7	0
4	smooth2	10	1
5	cube1	1	0
6	cube2	4	0
7	sphere1	4	0
8	sphere2	8	0
9	rough&cube	1	0
10	smooth&sphere	6	0
11	rough&sphere	2	0
12	smooth&cube	2	0

### Search Behaviour

**Figure 9:**
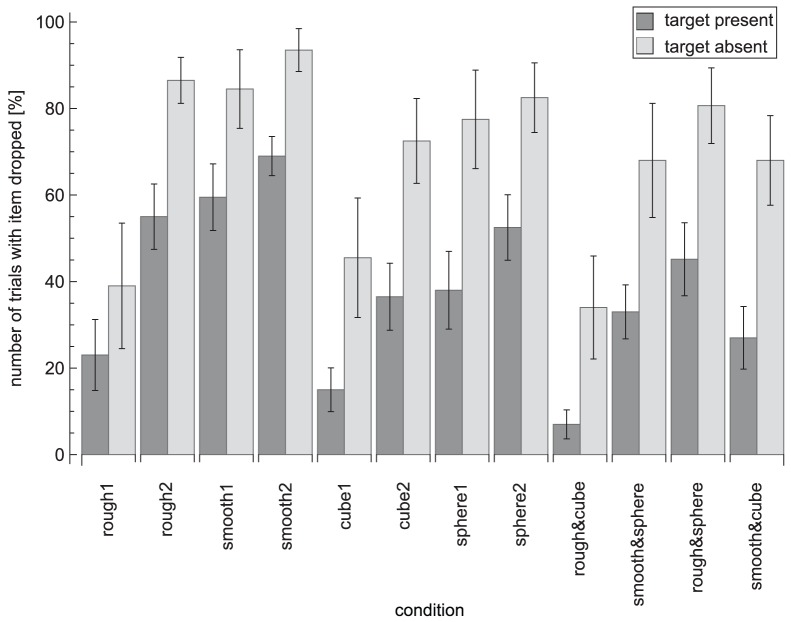
Proportion of trials in which items were dropped out of the hand for all conditions. Dark grey bars indicate target-present trials and light grey bars target-absent trials. Error bars represent standard errors.

In Figure 9 the proportion of times at least one item was released from the hand can be seen. This happened more often in target-absent trials than in target-present trials. The variability in exploration strategies between the conditions is also apparent in this graph, where more often an item is released in difficult search conditions. The results of the errors, the proportion of trials in which items were dropped out of the hand and the reaction times were mainly in line with each other (all *R*>0.80). This indicates that an increase in reaction time is accompanied by an increase in the number of errors and the number of times an item is dropped, which all reflect a decrease in search performance.

## Discussion

In the Introduction it was proposed that the performance in a search task is influenced by target identity, distractor identity and the difference between target and distractors. When search tasks with different targets or distractors are compared, this gives insight in how efficiently properties are processed and how they are integrated. The first aim was to confirm the saliency of roughness and shape. Next, we questioned whether these features might then also disrupt performance, even when they are irrelevant for the task. Finally, we investigated how shape and roughness can be integrated in the perception of objects and how cues and disruptions are balanced. In what follows, we will again discuss these research questions separately.

### Target Saliency

Previous research has shown that edges and vertices are salient compared to the absence of these features and that cubes are found faster amongst spheres than the other way round [4]. These results are replicated in this study. Reaction times were lower when participants had to search for the presence of a cube amongst spheres compared to the reversed situation. In addition, if a sphere was the target amongst cubes, more errors were made and more often items were dropped out of the hand. This exploratory behaviour suggests the use of a serial strategy by participants in this condition.

Likewise, Plaisier et al. [3] found that roughness was more salient than smoothness when patches of sandpaper on a display had to be explored. However, their set-up was 2D and the patches were presented against a background. This might have biased pop-out towards rough items compared to smooth, because these are more salient with respect to the smooth background (see also [21]). In our set-up, no background was present and the objects were 3D. It was found that the search for rough items amongst smooth distractors was faster than searching for smooth targets amongst rough distractors, which indicates that roughness is more salient than smoothness. In line with these results, fewer errors were made and less often items were dropped out of the hand in the search for the rough target. It must be kept in mind, though, that a fixed number of items was used in this study. So, no definite conclusions can be made about the change in reaction time with the number of items, which is an indication of pop-out. That is, a pop-out effect can only be established if the reaction time is independent of the number of items. It was not the intention of this study to thoroughly investigate whether roughness and edges were salient features, since this was already known [3], [4]. Still, these results suggest that the pop-out of roughness is a robust phenomenon. Noteworthy is that the optimal EP for the perception of roughness is lateral motion [8]. In our study, the participants had to initially grasp the bundle of items and lateral motions are more difficult with small items in the hand than if they are presented on a flat, stationary display. Still, participants were quite fast in the detection of the target.

In sum, in line with previous research it was found that targets that are rough and have edges are quickly found. In addition, a search asymmetry was found in favour of rough items compared to smooth items and shapes with edges to shapes without edges. This is in line with the idea that roughness and edges are salient features. This suggests efficient perceptual processing for roughness and edges. Next, this knowledge was used to manipulate the search conditions to investigate the influence of disruptions and the interactions of multiple salient and non-salient properties.

### Salient Disruptions

It was hypothesized that a property that can be salient would disrupt search performance, even if it were irrelevant to the search task. To investigate this, the presence of an irrelevant property that was added to both the target and distractors was compared to its absence. It was observed that the property disrupted performance in the search task, but not in all cases. If a search task was easy, then the addition of a salient irrelevant property was distracting; performance in the search for a rough item among smooth as well as a cube among spheres decreased when a disrupting property was present. This was seen in higher reaction times, more item drop behaviour and a larger number of errors. In contrast, when the task was already difficult, as was the case in the search for a sphere among cubes or a smooth item among rough items, no further significant decreases in performance were seen with the addition of a salient irrelevant property.

One possible influencing factor might have been that the manipulation of the items was hindered by the edges and roughness of the items; these items are more difficult to slide against each other, which would slow the reaction times. However, observations of participants’ behaviour indicated that this was not the case and that items could be easily moved in the hand. In addition, in the rough1 and cube1 conditions often no manipulation was performed and the initial grasp of participants was enough to give an answer. In the disrupted conditions, rough2 and cube2, more manipulations were performed and participants notably showed a more serial exploratory behaviour. Therefore, a more plausible explanation is that participants have to adopt a (more) serial strategy when irrelevant salient features are added to the search task. Possibly, they have to check whether a sharp sensation they feel is caused by a cube or by a rough item (or vice versa). In the difficult tasks, i.e. when a sphere or a smooth item is the target, the strategy is already serial and therefore task performance does not change notably.

From these results, it remains unclear how the distractors disrupt the perception of the target. One possible explanation is that the distractors add a lot of noise to the perception signal of the items (see also [22]). Hence, the signal-to-noise ratio of the target signal is decreased and the target is less easy to spot. Any top-down control to inhibit the noise of the distractors is apparently not strong enough to completely cancel this disruption and hence search performance decreases.

To summarize, both roughness and shape, in the form of edges or vertices, can disrupt performance, even when irrelevant for the task. The disruption of edges is consistent with the study of Panday et al. [11], who found that edges could diminish discrimination performance of aspect ratios in rectangular blocks. In search tasks, this disruptive nature of salient features was previously unknown. This stresses the notion that salient features cannot only improve search performance, but also decrease it.

### Integration of Target Cues

To investigate whether integration could take place between the two different object properties, conditions in which the target differed in two properties from the distractors were compared to conditions with only a single available cue. It was found that shape and roughness can be integrated and this leads to improvements in perceptual processing. The combined condition with a smooth sphere as a target among rough cubes was performed better than the search in conditions where only roughness or shape distinguished the target from the distractors. The differences were seen in a reduction of reaction times, fewer occurrences of a release of items out of the hand and fewer erroneous answers.

There is very little previous research into the integration of different object properties. Several studies have found (optimal) integration of separate cues that described a single object property [13]–[15], e.g. how force and position cues contribute to the perception of shape. These studies investigated discrimination thresholds, which were found to be lower in conditions where the two cues could be integrated. Conversely, other studies have shown that sometimes one cue is dominant over the other [16]–[18]. In the present study, the differences between object properties were well above just noticeable differences. Moreover, results show that integration can also take place between two different object properties instead of two cues that add up to a single object property. This suggests that the processing of different object properties is not completely independent. In this case there is no dominance of a single cue.

Another study that investigated integration of different object properties was that of Klatzky et al. [19] in a classification task. They showed the strongest integration for texture and hardness, but less integration between texture and planar contour. One of their arguments was that texture and hardness are more compatible in terms of EPs. In our experiment, the two EPs that are necessary to extract shape and roughness cannot be executed well simultaneously. Still, we find an integration of the two properties. Possibly an even more efficient integration can take place with two object properties in which the EPs are more compatible.

In contrast, not in all conditions with two cues an improvement to conditions with a single cue was seen. Possibly, for the fastest conditions (with smooth spheres as distractors), search was already quite efficient and the addition of a cue did not further improve performance. In other words, there might have been a floor effect for the reaction times in these conditions. In the mixed conditions, rough&sphere and smooth&cube, mixed results were found. When the target was a smooth cube among rough spheres, task execution improved compared to conditions with a smooth sphere as a target, but not compared to a rough cube. Perhaps in this case participants focused on the edges only and no integration took place. However, there was no significant difference between the two single cue conditions, smooth1 and cube2. The combined condition of rough spheres among smooth cubes did not show any improvements compared to the conditions with only one available cue.

To summarize these findings, it might be concluded that integration benefits the most if cues are non-salient. When one has to search for a non-salient target, this is usually a very inefficient search. When two non-salient cues are combined this extra information is used well and the search is much more efficient. To our knowledge, this is the first study that showed integration in a haptic search task. Future research could aim at investigating how this integration takes place. It is possible that the features are combined to search for a single conjunction target, or that one searches (simultaneously) for two properties until one of the two is felt.

### Cue and Disruption Balance

As described above, in some cases an extra cue can improve performance. However, sometimes the addition of a cue to a certain search task also results in the addition of a salient distractor property. This salient distractor, as discussed above, can disrupt task performance. Then, the question might be whether these expected enhancements (cues) and disruptions will balance each other out, or that one might weigh heavier than the other. This gives information about whether a feature is more “helpful” or more “disruptive”. Therefore, conditions with similar targets, but different distractors were compared. In this way, the target detectability with respect to its context can be examined. The number of cues is determined by the differences between the target and distractor and the number of disruptions by the number of salient properties in the distractors.

First, there were a few cases in which a cue was added to a certain condition, while at the same time a disruption was removed. As expected, in most cases the performance improved. This is not surprising, since the target in the combined conditions can be found using two cues. In addition, there is also one irrelevant salient property less in the distractors, so task efficiency is less disrupted. This is in agreement with the previous conclusions.

Secondly, to return to the balancing of cues and disruptions, another four comparisons in the data could be made. In these cases, a cue was added to a condition in combination with a disruption. The results were somewhat mixed, with sometimes improvements, but also decrements or no change at all. Perhaps the cues must be analysed separately. If so, it can be concluded that the addition of a shape cue accompanied by a shape disruption results sometimes in an enhanced performance. This means that the cue outbalances the disruption for the shape property. In contrast, the combined addition of a roughness cue and disruption sometimes gave a reduction in performance. This indicates that the disruption is weighted heavier than the extra cue for roughness perception. In other words, edges seem to be more beneficial than disruptive, whereas roughness appears to be more disruptive than helpful.

These interpretations are in line with the model that was made to describe the data. In this model, a linear regression was made to the data using four parameters and a constant. The parameters consisted of two cues and two disruptions, both for roughness and shape. Note that the absolute values of the weights do not have a real meaning, since the parameters were only fitted to the current dataset. The relative contributions of the parameters can, however, be compared. The weight of the shape cue was larger than that of the shape disruption. Also, the weight of the roughness disruption was larger than that of the roughness cue. In fact, the roughness cue was not even a significant weight factor. The model fitted the data well. This indicates that only 5 parameters are needed to explain 12 haptic search conditions. With other search tasks, the values of the parameters will of course be different, but still the search behaviour might be predicted with only a limited number of parameters, equal to the number of cues and disruptions.

A possible explanation for the weak roughness cue lies in the EP that is optimal for roughness perception. This EP is lateral motion [8] and might have been more difficult in a task where items were grasped and felt in the hand. The items move in the hand, which makes it harder to rub against it and perceive its roughness. The EP for shape is enclosure [8], which is easier in this task and might already be accomplished when participants grasp the bundle for the first time. Secondly, the shape cue might have been a bit stronger because it consists of several cues. The cube differed from the sphere in edges, vertices and curvature. In a similar search task, Plaisier et al. [4] showed that edges and vertices were the cues that best described the saliency of the cube.

### Conclusion

In conclusion, roughness and shape (i.e. edges and vertices) can be salient features. These features can enhance search performance when present in the target or disrupt task execution when present in the distractors. Roughness appears to be more disruptive than beneficial, whereas the reverse holds for shape. The balancing of cues and disruptions therefore seems to be related to the strength of the cue or disruption. If no salient features are present, different object properties can be integrated for greater performance. This is the first time this has been demonstrated in a search task.
